# Developmental bias of body size and caste by the fire ant social supergene

**DOI:** 10.1098/rspb.2026.1338

**Published:** 2026-10-07

**Authors:** Haolin Zeng, Corrie E. Eidson, D. Alekzandre Cushman, Mathews Nascimento, Beryl M. Jones, Brendan G. Hunt

**Affiliations:** ^1^ Department of Entomology, University of Georgia, Athens, GA, USA; ^2^ Odum School of Ecology, University of Georgia, Athens, GA, USA; ^3^ Department of Entomology, University of Kentucky, Lexington, KY, USA

**Keywords:** selfish genetic element, supergene, juvenile hormone, gas8, allosome, inversion polymorphism

## Abstract

Supergenes are clusters of linked genes that drive complex phenotypic divergence. In the fire ant *Solenopsis invicta*, a supergene determines polymorphic social organization, and its derived (*b*) haplotype is over-represented among large workers and gynes relative to the ancestral (*B*) haplotype, acting as a selfish genetic element. We tested whether this caste bias reflects altered juvenile hormone (JH) mediated growth by treating colonies with a JH analogue. We show that *BB* and *Bb* pupae reach similar maximal sizes and exhibit a comparable JH-mediated growth response despite consistent *Bb* over-representation in the upper size classes. We also used developmental gene expression data from another ant to reveal significantly enriched overlap between genes with differential expression by supergene genotype and orthologues with differential expression between gyne- and worker-destined larvae. This overlap could be shaped in part by observed *cis*-regulatory evolution of the supergene. Overall, our results are consistent with incompletely penetrant effects of a supergene on developmental trajectories linked to size-based caste allocation in a highly social insect.

## 1. Introduction

Chromosomal inversions and other genome structural variants can facilitate the evolution of complex trait polymorphisms by ensuring sets of coadapted alleles remain genetically linked in large haplotype blocks known as supergenes [1,2]. Across diverse taxa, supergenes have been causally linked to alternative morphs, mating strategies and reproductive behaviours, despite the potential to accumulate deleterious mutations and exhibit recessive deleterious effects [3]. In some cases, derived supergene haplotypes are less susceptible to such fitness deficits because they act as selfish genetic elements and bias their own transmission relative to those not carrying the haplotype, a feat that can be achieved through myriad mechanisms [4]. In eusocial insects such as ants, supergenes can exploit both individual phenotypes and colony social organization to promote their own transmission [5–7].

At least six distinct ant lineages possess independently evolved supergenes that control queen number [8,9], as well as additional aspects of individual and colony natural history that are best documented in two ant genera: *Formica* and *Solenopsis* [6]. In *Formica* ants (subfamily Formicinae), biased supergene transmission occurs through maternal effects [10] and assortative mating [11]. In *Solenopsis* ants (subfamily Myrmicinae), biased supergene transmission has been documented through enhanced transmission of the derived haplotype to reproductive individuals via a combination of developmental effects [12,13] and adult greenbeard discrimination [5,14,15].

The *Solenopsis* supergene arose relatively recently (around 1 Ma [16], compared to 20–40 Ma in *Formica* [17]) and spread to multiple congeners via introgression [18,19]. The derived *Social b* chromosome haplotype differs from the ancestral *Social B* chromosome (hereafter simplified as *b* and *B*) by the presence of three linked chromosomal inversions and a linked centromeric region of suppressed recombination, collectively forming a single supergene that spans over 500 individual genes [20,21]. Beyond the well-known regulation of colony social structure, whereby *b* haplotype-carrying colonies house multiple egg-laying queens, the supergene also shapes a suite of ontogenetic and life-history traits in ways that appear paradoxical (as is often the case for selfish genetic elements): although *b*-carriers often exhibit reduced individual performance or viability, the haplotype nonetheless enhances its own transmission [22,23].

On one hand, the *b* haplotype is associated with inferior reproductive phenotypes. Winged queens with the *BB* genotype possess greater fresh weight, fat reserves and dispersal capacity, and after mating, they are more successful at founding colonies without workers than queens with the *Bb* genotype [24–27]. By contrast, *Bb* queens rely on existing worker support by joining mature polygyne colonies [24,26]. Moreover, homozygous *bb* individuals exhibit exceptionally low weight and reproductive potential [25,28,29], and haploid *b* males are known to attain smaller body sizes and lower sperm counts than *B* males [13,30].

On the other hand, within polygyne colonies, the *Bb* genotype is over-represented relative to the *BB* genotype in pre-reproductive queens (gynes) [12,31], and the *b* haplotype is over-represented in haploid (reproductive) males [13]. These biases occur already at pupation [12,13], before the onset of additional greenbeard-mediated culling of adult gynes by workers [5,32]. Moreover, the *Bb* genotype is over-represented relative to *BB* in large adult workers compared to small adult workers [12,33]. Thus, the *b* haplotype probably exerts an effect on individual ontogeny to pleiotropically produce an over-representation of *b*-carrying individuals in the large worker and reproductive castes.

This project aims to document the developmental mechanisms underlying effects of the fire ant social supergene on body size and caste allocation. In conceiving the study, we posited that supergene effects on the coordination of developmental processes may be mediated by evolutionary changes to the signalling or reception of growth hormones important to social insect caste determination and differentiation [34–36], paralleling endocrine control of alternative mating morphs observed in avian supergenes [37,38]. Although invertebrates and vertebrates differ vastly in physiology and life history, their supergenes may similarly act as master regulators of morphological differentiation by modulating hormone-driven developmental pathways [39].

Here, we focus on juvenile hormone (JH), a central endocrine regulator in insects that influences both body size and caste fate. During the larval stage of development in a holometabolous insect (characterized by larval, pupal and adult life stages), JH suppresses moulting of the exoskeleton (ecdysis), thereby potentially extending growth periods [40,41]. Small differences in JH titer or sensitivity during critical developmental windows can be amplified to produce large differences in final body size. In social insects, these JH-mediated timing effects also intersect with nutritional regimes and social cues to shape caste differentiation, contributing to the divergence between non-reproductive (worker) and reproductive (gyne) phenotypes [42,43]. Experimental treatments of JH and JH analogues in ants have resulted in increased larval growth and upwardly shifted body-size distributions, and could bias individuals toward gyne or worker subcaste differentiation [34,44–51]. Thus, JH represents a potent axis through which genetic variation at the social supergene could be translated into alternate developmental phenotypes, possibly by altering hormone sensitivity or shifting the temporal windows over which JH acts.

We tested whether the social supergene biases body size and caste through a combination of experimental perturbation and comparative gene regulatory analysis. First, we employed dietary application of a JH analogue to whole colonies containing mixed-age broods to probe whether the supergene modulates developmental outcomes such as body size (measured as pupal weight) and caste fate via major differences in JH sensitivity [52,53]. Second, we used previously published supergene-associated gene regulatory and expression datasets [54–57] and caste-associated developmental gene expression profiles from another ant from the subfamily Myrmicinae [49,58] to identify potential overlap between supergene-induced, JH-induced and caste-biased transcriptional changes. Our results highlight how supergenes bias complex life-history traits and implicate gene regulatory evolution as a potentially important component of supergene effects on developmental variation.

## 2. Material and methods

### (a) Ant collection and experimental procedure

Queenright and mature colonies of *Solenopsis invicta* representing both social forms were collected from Clarke and Oconee Counties, Georgia, USA, in the spring of 2024. After establishing colonies in the laboratory, we set up queenless experimental units from each social form (figure 1A), with each unit receiving 1.5 g of workers (approx. 2500 individuals) and 1.5 g of mixed brood, both obtained from a distinct, mature queenright source colony (hereafter referred to as the ‘source colony’). For each social form, a matched pair of acetone-control and methoprene-treated units was established on the same day, using brood and workers from the same source colony. Methoprene treatment (Sigma-Aldrich, catalogue no. 33375, CAS No. 40596-69-8) or acetone control diet was administered once, one day after colony set-up. This feeding approach allowed methoprene to reach all developing larvae via workers. We treated mixed-stage brood because caste determination in *S. invicta* may occur early in larval or embryonic development [59,60]. All experimental colonies were maintained under standardized rearing conditions and were subsequently fed ad libitum with untreated alternating diets (see the supplementary material for details).

**Figure 1.**
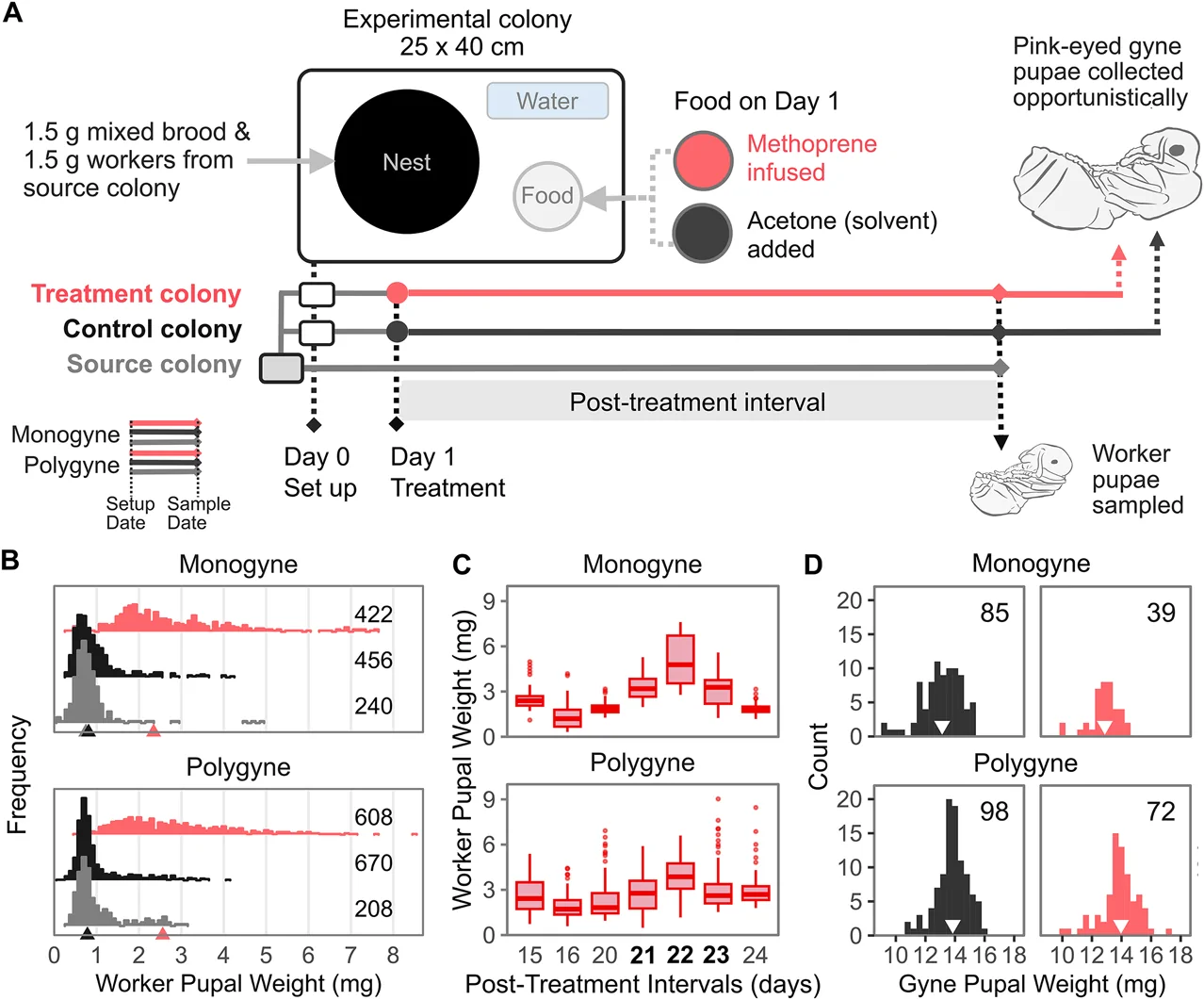
Experimental design for studying growth-related effects of methoprene (a JH analogue) treatment on worker and gyne pupal weight in *Solenopsis invicta*. (A) Overview of experimental set-up. Each experimental colony was assembled by combining workers and mixed-age brood from a source colony into a plastic tray. Methoprene exposure occurred only once; colonies were subsequently fed untreated food for the remainder of the post-treatment intervals. For each designated post-treatment interval, paired monogyne and polygyne colonies were established simultaneously. (B) Distribution of all collected worker pupal weights (measured to 0.01 mg) by treatment and social form. Numbers indicate total pupae sampled. Triangles indicate median weights. (C) Boxplots of worker pupal weight by post-treatment intervals from methoprene-treated colonies. (D) Histograms of gyne pupal weights by treatment and social form. Numbers indicate total gyne pupae sampled. White triangles indicate median weights.

To thoroughly assess developmental trajectories under methoprene treatment, we varied the number of days between treatment initiation and pupal collection, which was defined as the ‘post-treatment interval’, including 15, 16, 20, 21, 22, 23 and 24 days. We included an additional polygyne pair for a 25-day post-treatment interval to receive more extra-large worker pupae for comparative analysis within the polygyne social form. For each interval, we simultaneously established two treatment–control pairs, one pair sourced from a unique monogyne colony (*n* = 7) and one pair sourced from a unique polygyne colony (*n* = 8).

From each polygyne unit, we collected and weighed 96 worker pupae (except for 24-day methoprene-treated units, where fewer pupae remained). From each monogyne unit, 50–60 worker pupae were collected and weighed. We also weighed 30 haphazardly selected worker pupae from each corresponding source colony on each collection day. Pink-eyed pupae of gynes were sampled from the units as they appeared, from as early as 19 days to as late as 51 days after treatment.

We genotyped polygyne pupae from all experimental colonies for markers of supergene genotype [61,62], targeting worker pupae in the upper and lower 25% weight quantiles to efficiently sample a broad range of pupal sizes. To more fully evaluate differences in size distributions by genotype, we genotyped all the worker pupae collected from the 21-, 22- and 23-day post-treatment units, as these produced the largest pupae (figure 1C), as well as all sampled gyne pupae. In total, we genotyped 1049 worker pupae and 195 gyne pupae from polygyne-derived experimental units (monogyne-derived experimental units did not require genotyping, as all individuals are *BB*).

We note that multiple-comparison correction was not applied to weight comparisons because each statistical test addresses a distinct, pre-specified hypothesis, rather than representing exploratory pairwise contrasts within a larger family.

### (b) Gene expression analysis

Previously, we performed integrative analyses [56] of *S. invicta* chromatin accessibility (ATAC-seq), adult gene expression (RNA-seq) [53,55], *B* and *b* single nucleotide polymorphism (SNP) allele frequencies [62], and transcription factor binding motifs [56]. We were further interested in comparing these data to developmental patterns of gene expression affiliated with the differentiation of body size and caste, as well as the regulatory response to methoprene treatment, but such data are currently unavailable for *S. invicta*. Fortunately, an emerging model of ant development, *Monomorium pharaonis*, belongs to the same subfamily (Myrmicinae) as *S. invicta* and has been subject to an examination of methoprene-responsive and caste-biased gene expression during larval development [49]. A summary table compiling accession numbers, per-dataset mean read depth or mapping rate and additional quality metrics is provided in the supplementary material, table S1.

We identified orthologues between *S. invicta* and *M. pharaonis* using a BLAST [63] reciprocal best hit approach (*n* = 9884; supplementary material, table S2) to examine overlap between sets of genes with (i) evidence of caste-biased or JH analogue-responsive gene expression during larval development in *M. pharaonis*, and (ii) evidence of gene regulatory evolution and differential expression in multiple tissues of adult *S. invicta.* We first identified genes that were supergene-biased in expression in *S. invicta* (i.e. *Bb* > *BB* in four contexts: worker brains, worker gasters (abdomen), gyne brains and gyne ovaries (all adults)) (supplementary material, table S3). We next tested for overlap of these genes with orthologues identified as gyne-biased (relative to workers) or responsive to JH analogue treatment (relative to controls) during larval development. We considered genes for which expression in *M. pharaonis* was gyne-biased in all three larval stages tested (early, middle and late 3rd instar (the final larval instar)), or JH-biased in at least one of the three larval stages tested (since no genes were consistently affected by JH across all 3rd instar stages; supplementary material, table S3) [49]. We tested the significance of resulting overlaps using the SuperExactTest package in R (supplementary material, table S4) [64,65].

## 3. Results

### (a) Overall methoprene effect

Our experiments aimed to examine how the fire ant supergene influences pupal size in the presence and absence of JH-analogue (methoprene) treatment. In polygyne-derived experimental colonies, worker pupal weights from acetone-control groups (overall median weight = 0.77 mg, *n* = 723) did not differ significantly from those in their corresponding source colony pupae (median weight = 0.80 mg, *n* = 238; Mann–Whitney *U*-test, *p* = 0.398; figure 1B). Although a small but statistically significant increase was observed in monogyne acetone-treated pupae (*n* = 456) compared to source colonies (*n* = 240, Mann–Whitney *U*-test, *p* < 0.001), the absolute difference was modest (median weight: 0.81 versus 0.74 mg) and the effect size was small (rank-biserial correlation = 0.13). Methoprene increased expected worker pupal weight by 2.6-fold compared with the acetone control pupae (gamma generalized linear mixed model (GLMM) with log link, colony as a random effect: *β* = 0.935, *z* = 28.61, *p* < 0.0001; figure 1B), with a 3.2-fold difference in overall median weight (2.51 versus 0.78 mg). Social form alone did not affect worker pupal weight (gamma GLMM, *p* = 0.724), and the methoprene response did not differ significantly between social forms (gamma GLMM, treatment × social form: *p* = 0.224).

Pupal weights from the treated colonies showed a temporal trend across post-treatment intervals, with weights increasing to a peak at the interval of 22 days and declining thereafter (figure 1C). This pattern was consistent in both social forms. The observed trajectory confirmed that our experiment captured a broad range of growth responses under a single treatment of methoprene owing to the temporal structure of the experimental design.

We maintained the colonies beyond the sampling period of worker pupae because gyne pupae continued to emerge (until as late as 51 days post-treatment). In both social forms, methoprene treatment significantly reduced the number of gyne pupae produced (Poisson generalized linear model (GLM) on colony-level counts: treatment effect, *p* = 0.004). However, methoprene treatment did not significantly affect gyne pupal weight (figure 1D). During this extended period of gyne collection, we also observed many unusually large workers in methoprene-treated colonies that resembled intercastes (supplementary material, figure S1; [59]).

### (b) Worker pupal weight and genotypic pattern

To determine whether individuals respond differently to methoprene treatment by supergene genotype, we first genotyped and compared all sampled pupae from post-treatment intervals of 21–23 days, which showed the greatest overall growth response (figure 1C). *Bb* pupae did not differ significantly from *BB* pupae in weight in the merged day 21–23 acetone-control data (median: 0.77 versus 0.74 mg, Mann–Whitney *U*-tests, *p* = 0.072) or in the equivalent methoprene-treated data (median: 3.16 versus 2.79 mg, Mann–Whitney *U*-test, *p* = 0.055; figure  2A). The median normalized methoprene-to-acetone weight ratio also did not differ significantly between the genotypes (median: 4.24 versus 3.78 for *Bb* and *BB*, Mann–Whitney *U*-test, *p* = 0.22; figure 2B). Nevertheless, the proportion of *Bb* individuals in the same dataset increased gradually from approximately 50% in the lowest weight quartile to approximately 70% in the highest quartile, corresponding to a significant over-representation of *Bb* pupae among larger size classes relative to the small-size class in both the methoprene treatment and acetone control groups (figure 2C).

**Figure 2.**
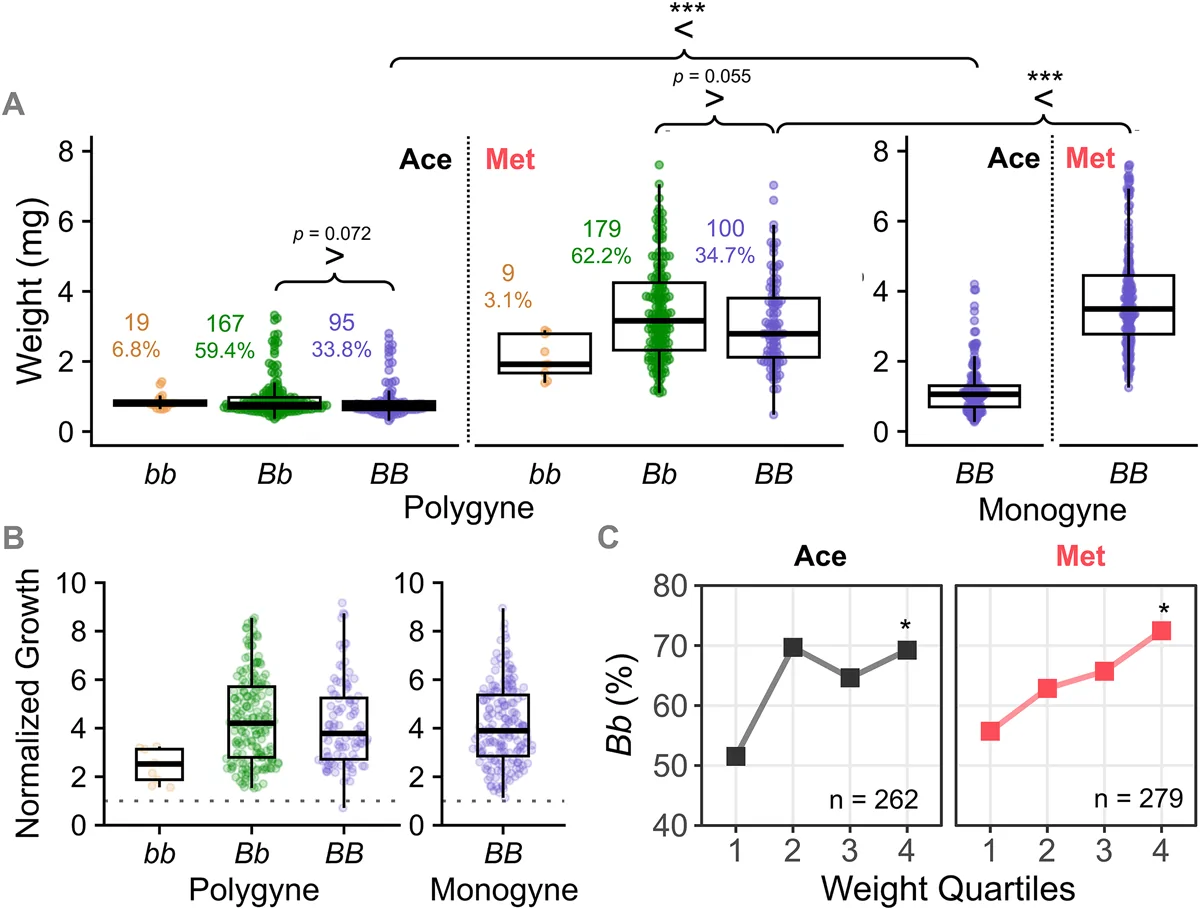
Worker pupal weight distributions and genotype enrichment across treatments. Data include post-treatment intervals of 21–23 days where all collected pupae were genotyped. (A) Distributions of worker pupal weights. (B) Boxplots of normalized methoprene response, where points indicate individual methoprene-treated pupal weight relative to the acetone control colony median. (C) *Bb* genotype frequency across weight quartiles. Asterisks denote statistical significance at the 0.05 (*) and 0.001 (***) levels based on Mann–Whitney *U*-tests comparing social forms (A) or chi-square tests comparing the bottom (first) and top (fourth) quartiles (C). Marginal *p*-values of 0.05–0.1 are shown in panel (A).

We then examined all post-treatment intervals, where all pupae from the top and bottom quartiles by weight were genotyped. Overall, *BB* pupae were observed to be significantly lighter than *Bb* (19% lower; gamma GLMM, *p* = 0.008), but the methoprene response did not differ significantly by genotype (treatment × genotype: *β* = 0.085, *z* = 0.91, *p* = 0.36). *BB* individuals were less likely than *Bb* to occur in the top quartile versus the bottom quartile by weight (binomial GLM, significant effect of genotype, *β* = −0.60, *z* = −2.30, *p* = 0.02). In this binomial GLM, methoprene treatment again had no significant effect (*p* = 0.44), and the treatment × genotype interaction was also not significant (*β* = 0.37, *z* = 1.03, *p* = 0.30). In addition, the proportion of *Bb* worker pupae in the top or bottom weight quartiles did not differ between methoprene-treated and acetone-control groups (Mann-Whitney tests, *p* > 0.9; supplementary material, figure S3). Together, these results suggest that methoprene treatment did not disrupt an underlying size-genotype association established during development.

Despite the enrichment of *Bb* we observe among large worker pupae, *Bb* and *BB* individuals overlapped extensively across their overall weight distributions (figures 2A and 3; supplementary material, figure S2). To compare the upper size limits of *BB* and *Bb* pupae, we bootstrapped the top 5% weight for each genotype (10 000 resamples) and computed 95% confidence intervals (CI) on the difference. Under both conditions, observed differences were small (acetone: −0.13 mg, 95% CI: −0.63, 0.32; methoprene: −0.09 mg, 95% CI: −0.66, 1.14) and CIs spanned zero. Thus, we found no indication that the maximal size of *Bb* pupae could exceed that of *BB*.

**Figure 3.**
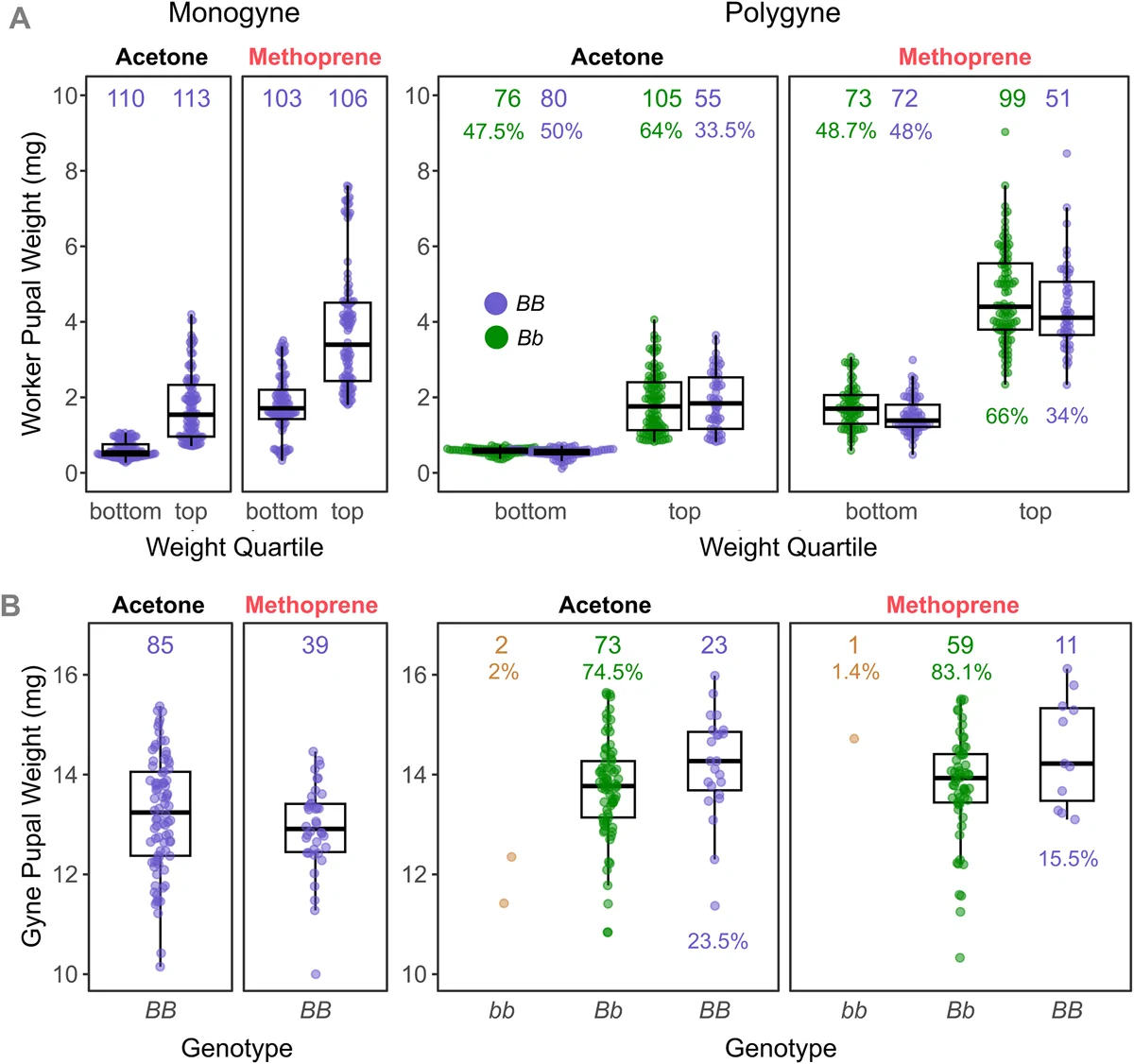
Pupal weight distributions by genotype, social form and treatment within large (top quartile) and small (bottom quartile) size classes for workers (A) and gynes (B). Numbers above each group indicate sample size, and percentages denote the proportion of each genotype (among all three genotypes). Data aggregated from post-treatment intervals of 15–24 days. For worker pupae (A), no significant differences in weight were detected between *BB* and *Bb* within the same treatment and social form, nor between *BB* weights across social forms within the same size class (gamma GLMMs). *bb* worker pupae are omitted. For gyne pupae (B), weights did not differ significantly between polygyne *BB* and *Bb* individuals, but polygyne *BB* individuals were significantly heavier than monogyne *BB* individuals under both acetone and methoprene treatments (Mann–Whitney *U*-tests).

We found 3% *bb* worker pupae among all genotyped samples in the current study (32 out of 1049 pupae). Similar to previous findings that *bb* individuals have exceptionally low weight [24,33], and that haploid *b* males are smaller than haploid *B* males [13], none of the *bb* worker pupae we sampled exceeded 3 mg in weight, even under methoprene treatment (median weight was 0.94 mg in methoprene-treated *bb*, compared with 2.34 mg and 2.62 mg in *BB* and *Bb* worker pupae, respectively). These results add additional evidence that the *b* supergene is recessive and deleterious [29] and has dramatic effects on growth potential for *bb* larvae.

Although there was no effect of social form on methoprene-mediated growth, we detected an effect of social form on *BB* pupal weight. For the worker pupae from post-treatment intervals that were fully genotyped (21–23 days), monogyne *BB*s weighed more than the polygyne *BB*s in both the acetone-control (median: 1.06 versus 0.74 mg) and the methoprene-treated groups (median: 3.50 versus 2.79 mg; Mann–Whitney *U*-tests, *p* < 0.001; figure 2A). However, this effect was not apparent in normalized growth responses (figure 2B) or when all post-treatment intervals were considered (figure 3). An opposite trend was observed for gyne pupal weight: polygyne *BB* gyne pupae were significantly heavier than monogyne *BB* gyne pupae under both control (Mann–Whitney *U*-test, *p* < 0.001) and methoprene-treated conditions (*p* < 0.001; figure 3B).

### (c) Gyne pupal weight and genotypic pattern

The *Bb* genotype was over-represented among gyne pupae in both acetone-control and methoprene-treated groups and showed stronger enrichment than in workers (overall *Bb* frequency, acetone-control colonies: gyne 73.5% versus workers 56.8%; methoprene-treated colonies: 80.8% versus 56.6%).

When worker and gyne pupae were analysed together along the same size axis, the increase in *Bb* frequency with body size was observed across castes (binomial GLM, effect of weight, likelihood-ratio *χ*² = 26.56, *p* < 0.001; figure 4). The slope of this increase did not differ significantly between castes (caste × weight interaction: *p* = 0.73) or between control and treatment (treatment × weight interaction: *p* = 0.23), and no higher-order interactions were detected. This pattern suggests that *Bb* enrichment at large sizes is owing to a shared size-linked developmental bias by the *b* supergene rather than a direct caste trade-off.

**Figure 4.**
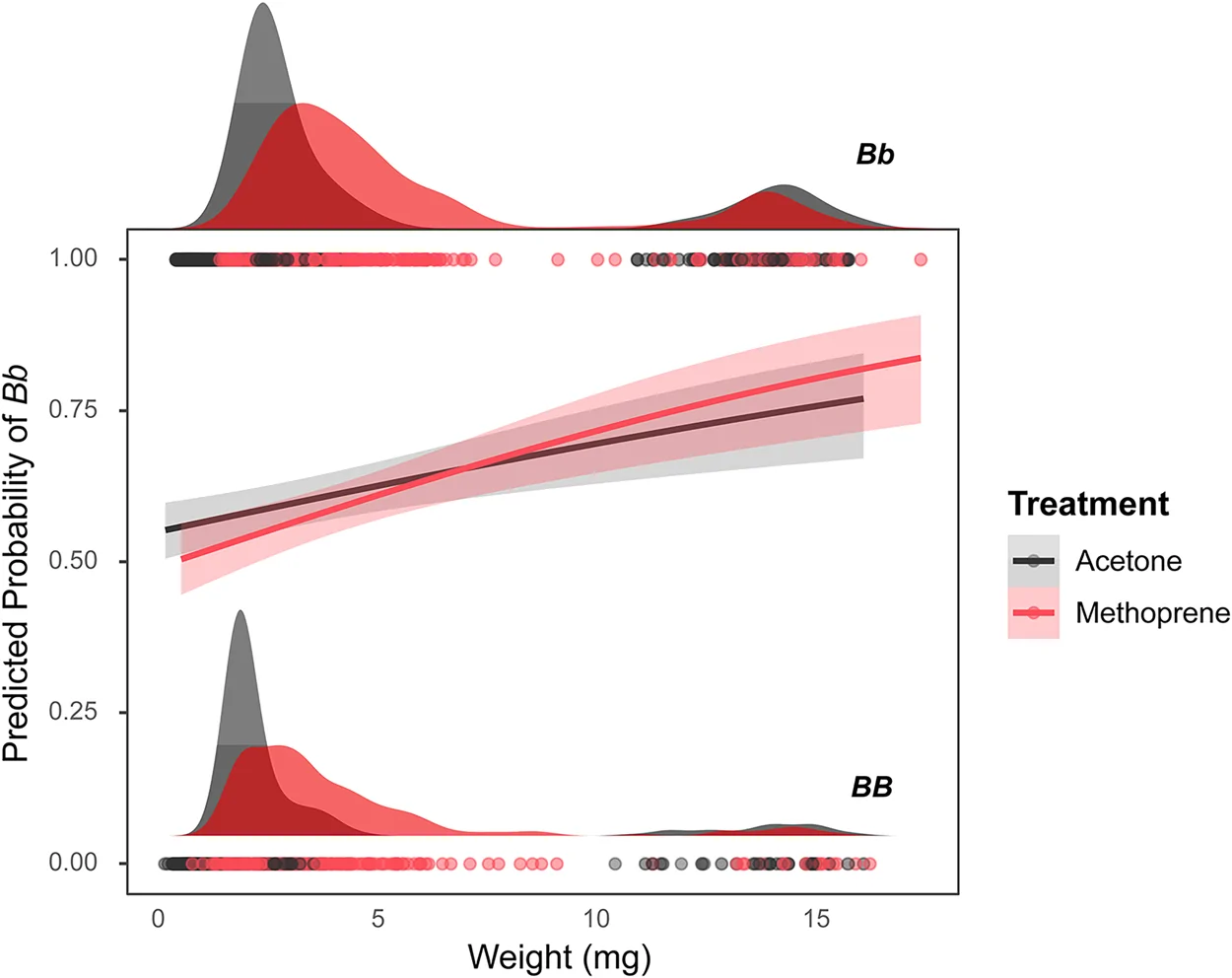
Probability of the *Bb* genotype by pupal weight increases across castes but is not significantly altered by methoprene treatment. Curves show binomial logistic regressions with 95% confidence intervals, and density ridges indicate weight distributions for each treatment. The *bb* genotype was omitted.

### (d) Overlap of genes involved in caste development, hormone response and supergene evolution

We hypothesize that the observed association between supergene genotype and body size may be driven by supergene-mediated changes in the regulation of genes implicated in caste development in ants. Modification of growth trajectories related to adult caste development occurs during the larval or perhaps even embryonic stages, but currently there are no comprehensive gene expression studies of caste development in *S. invicta.* Thus, we used data from an emerging model of ant development in the same subfamily, *M. pharaonis*, to test for overlap between gene sets with caste-biased gene expression during larval development [49] and differential expression by supergene genotype in *S. invicta* adults [54,56].

If the supergene modifies development such that *b* carriers exhibit a more gyne-like developmental trajectory, and gyne-biased expression patterns are conserved across species, we would expect supergene-biased genes to overlap with gyne-biased genes. Among 9231 predicted orthologous gene pairs with expression data in both species across studies, 1076 genes exhibit constitutively elevated expression in gynes during the 3rd larval instar (early, middle and late-stage 3rd instar) relative to workers in *M. pharaonis* [49]. Comparing these gyne-biased genes to 191 genes previously identified as upregulated in *Bb* carriers across adult caste and tissue contexts in *S. invicta* [54,56,57], 34 (18% of *Bb-*biased genes) overlapped, significantly more than expected by chance (fold enrichment (FE) = 1.53, *p* = 0.007). Strikingly, one gene is *Bb-*biased in all adult *S. invicta* contexts (adult worker brain, worker gaster, gyne brain and gyne ovary) and also biased in gynes throughout early, middle and late 3rd instar larval development in *M. pharaonis* (figure 5; FE = 6.16e + 06, *p* = 6.49e-07). This gene, *growth arrest specific protein 8* (*gas8;* LOC105199315), is notably located within the fire ant supergene.

**Figure 5.**
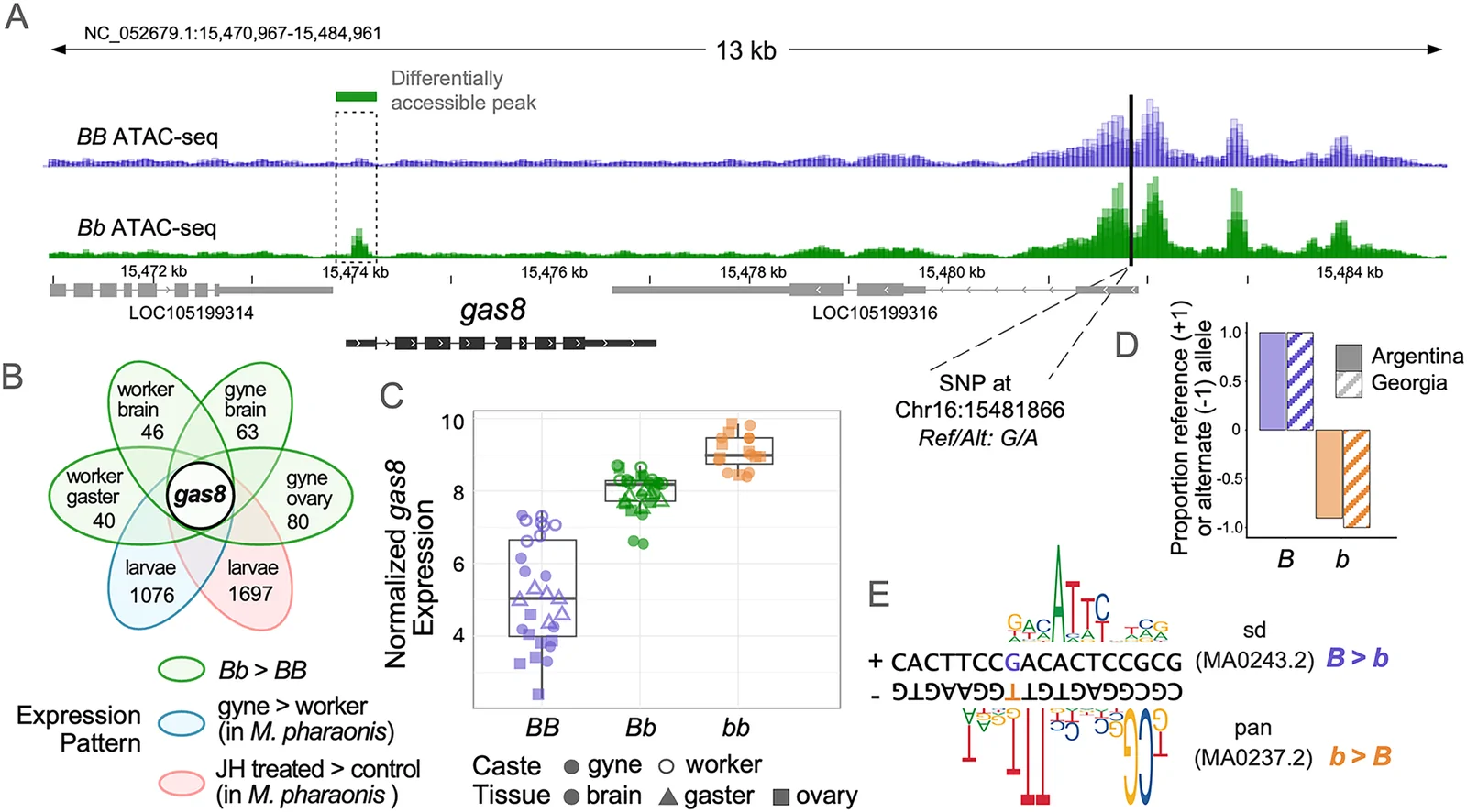
Chromatin accessibility, expression patterns and *cis*-regulatory divergence of *gas8* between social chromosome haplotypes. (A) Adult worker brain ATAC-seq coverage across the *gas8* genomic region reveals haplotype-specific peaks within the gene, indicating differential chromatin accessibility by supergene genotype. Gene models (untranslated regions (UTRs), exons and introns) are shown below tracks for reference. (B) Gene expression summary compiled from multiple RNA-seq datasets; *gas8* (LOC105199315 in *S. invicta* or LOC105835368 in *M. pharaonis*) was the only gene that overlapped all comparisons. ‘Gyne > worker’ comparison includes differential expression across early, mid and late third-instar larvae, whereas ‘JH treated > control’ includes any third-instar stage. (C) Individual-level RNA-seq expression of *gas8* in *S. invicta* shows consistently higher expression in *Bb* adult tissues (green) than in *BB* adult tissues (purple). (D) A nearly-fixed SNP between *B* and *b* (allele frequency data from [66]) is located downstream of *gas8* and in the UTR of *Fut2* (LOC105199316), which influences predicted binding of transcription factors shown in panel (E). (E) Transcription factor binding motifs overlapping the SNP downstream of *gas8* show haplotype-biased predicted binding affinities. Motifs occur on opposite strands within ATAC-defined peaks within a footprint indicative of transcription factor binding.

We additionally examined the overlap between gene sets with supergene-biased expression in *S. invicta* and JH-responsive expression in *M. pharaonis.* Characterization of experimental individuals in *M. pharaonis* revealed that JH treatment of worker-destined larvae induced more gyne-like phenotypic and transcriptomic characteristics [49], suggesting that JH regulates growth pathways also involved in caste differentiation. Although the absence of a genotype-specific growth response to JH in our study is not consistent with large differences in JH sensitivity by genotype, we cannot rule out supergene action on downstream growth and caste-regulatory pathways that partially overlap with JH-responsive networks. Since *Bb* biases development towards both gyne production and increased size in *S. invicta,* we predicted that the supergene may also act on these shared pathways. In total, 1697 genes showed elevated expression in response to JH treatment during the third instar larval stage in *M. pharaonis* [49], and 47 of these genes are also more highly expressed in *Bb* carriers in *S. invicta* (FE = 1.34, *p* = 0.024). In addition, all but eight of the shared JH-responsive and *Bb-*biased orthologous genes are also upregulated in gynes (FE = 2.95, *p* = 1.66e-09), including *gas8*. Importantly, while *gas8* is gyne-biased across 3rd instar larval development of *M. pharaonis,* this pattern changes later in development, with no significant difference in pre-pupae gynes versus workers (adjusted *p* = 0.41 [49]) and worker-biased expression in young pupae (log2 fold change (FC) = 1.94, adjusted *p* = 2.83e-12, [49]). These results are consistent with the hypothesis that the supergene has evolved to modify existing hormone-mediated growth and caste differentiation pathways to bias representation among reproductive individuals.

Because *gas8* emerged as a candidate gene for supergene effects on biased growth in supergene carriers based on our transcriptomic analyses, we further investigated the evidence for supergene-mediated gene regulatory evolution of this locus in *S. invicta*. Chromatin accessibility analysis of *gas8* via ATAC-seq profiling in adult worker brains [57] revealed multiple regions of open chromatin around the *gas8* locus, with chromatin accessibility elevated in the 5’ untranslated region (UTR) of the gene in *Bb* as compared to *BB* (figure 5A). This increased accessibility is consistent with elevated expression of *gas8* observed in *Bb* compared with *BB* individuals in adult gynes and workers (figure 5C) [56]. In addition, a previous analysis in *S. invicta* of allele-specific expression in heterozygous adult gynes (*Bb*) found that *gas8* was among the loci with significant *b* allele-biased expression [54]. These results suggest the supergene has evolved to modify the activity of *gas8* across contexts, which may play a role in altering the developmental trajectory of supergene carriers.

To investigate potential sequence divergence underlying supergene-mediated regulatory effects on *gas8* expression, we examined supergene haplotype-specific variation in predicted transcription factor binding motifs [57]. Two key motifs were identified within a 10 kb window of the *gas8* locus with significant differences driven by a nearly-fixed SNP observed between *B* and *b* (figure 5D): sd (Scalloped), with stronger predicted binding in the *B* haplotype (*B* > *b*), and pan (Pangolin), with stronger predicted binding in the *b* haplotype (*b* > *B*; figure 5E). The location of this putative transcription factor binding site coincides with a ‘footprint’ in an otherwise open chromatin peak, a general feature of chromatin accessibility at bound transcription factor sites [67]. In addition to being downstream of *gas8,* this locus is also within the 5’ UTR of LOC105199316 (*galactoside alpha-*(*1,2*)*-fucosyltransferase 2,* or *Fut2*)*. Fut2* is more highly expressed in *Bb* compared to *BB* worker gasters (log2FC = 2.05, adjusted *p* = 2.80e-15), but is not supergene-associated in other adult contexts we examined (supplementary material, table S3). Intriguingly, *Fut2* exhibits supergene-linked copy number variation, with greater copy number in *B* compared with *b* [55,57]. In addition, *Fut2* expression is worker-biased (compared with gyne) throughout 3rd instar larval development in *M. pharaonis* [49] (supplementary material, table S3), a pattern opposite to that of *gas8*. Whether regulatory divergence in this region contributes to supergene-associated effects on growth and caste determination in *S. invicta* should be examined in future studies, but our results provide compelling evidence that this locus may be a target of supergene *cis*-regulatory evolution.

## 4. Discussion

In *S. invicta*, the social supergene influences a broad suite of adult traits, yet its developmental effects at the larval stage, particularly those that may underlie the over-representation of supergene carriers (genotype *Bb*) among large workers and gynes in polygyne colonies, remain largely unexplored. Our study shows that the social supergene is associated with coordinated shifts in both caste outcome and body size [36]. *Bb* individuals were disproportionately represented among large worker and gyne pupae, and methoprene shifted the overall weight distribution upwards without significantly altering the direction or magnitude of this genotype bias (figure 4). This pattern parallels previous findings of the same pattern of size bias by genotype in adult workers (figure 6) [12,33,70]. Because we found no evidence that *Bb* individuals attain a larger maximal size than *BB* individuals, these results point to a complex developmental origin of supergene-associated size bias.

**Figure 6.**
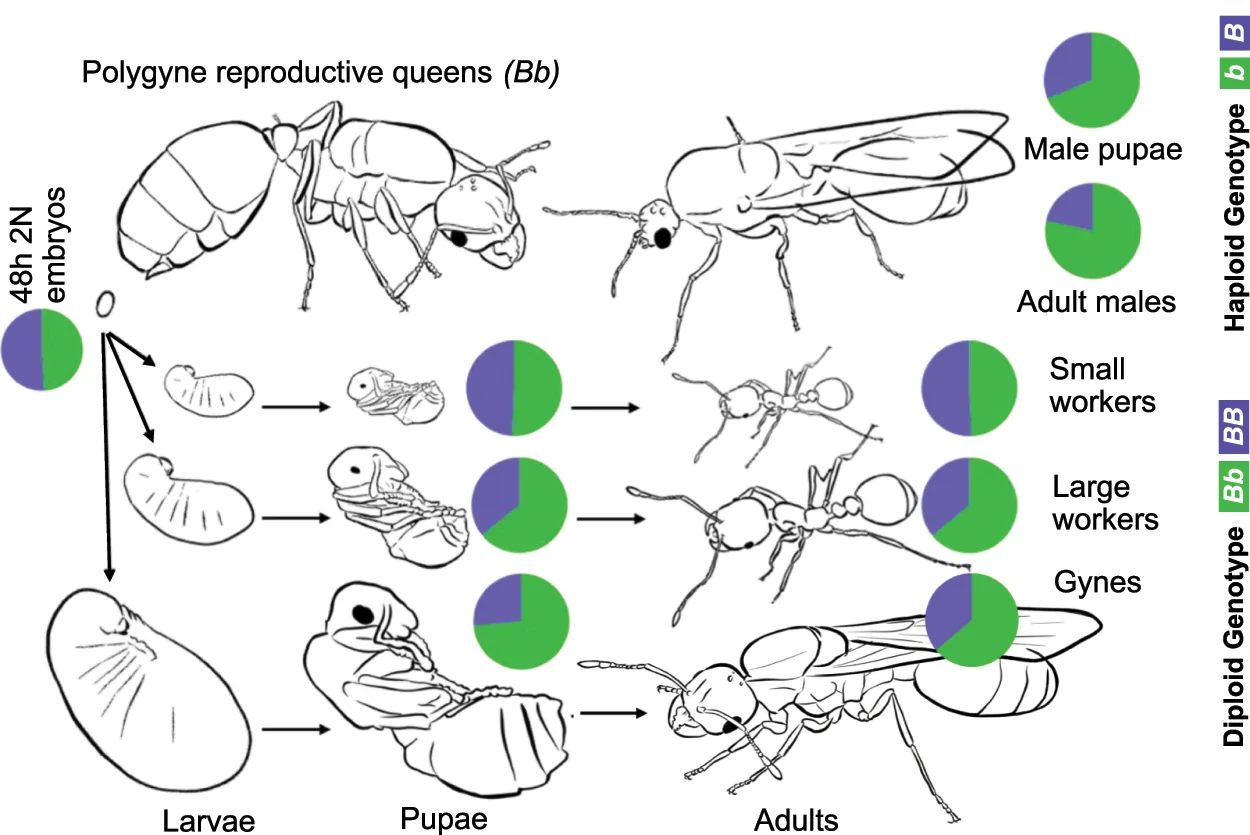
Current knowledge on patterns of genotype frequency across castes and life stages in polygyne *S. invicta* from Georgia, USA. These estimates are compiled from multiple published sources (early diploid embryos [68]; pupal and adult males [13]; adult workers [12]) and the present study (worker and gyne pupae data are from acetone-control colonies for which we do not know the corresponding embryonic genotype frequencies; see the supplementary material for adult gyne data). All reproductive queens in invasive polygyne populations are *Bb* [5,28,31]. The *bb* genotype is omitted for visual clarity. Note that progeny in [12,68] were derived from queens singly mated to haploid *B* males, which is the predominant mating pattern [31], and that most mature adult *BB* gynes are subject to culling by *Bb* workers before nuptial flights in polygyne colonies [5,32,69].

The overall *Bb* enrichment among offspring we observed for polygyne colonies (57% *Bb* versus the Mendelian expectation of 50%, binomial test, *p* < 0.001) could be explained by the loss of *bb* larvae owing to recessive deleterious effects [20,31,54] (*b* mating frequency is limited and dependent on local polygyne colony density [23]). Under breeding-system parameters from a Georgia population [31], eliminating all *bb* offspring would yield an expected *Bb* frequency of roughly 53%, and a similar calculation for a Florida population yields 55%. These values closely match the pooled *Bb* proportion of 57% observed in our worker pupal dataset. We nevertheless detected *bb* in 3% of worker pupae, indicating the lack of completely penetrant larval lethality for *bb*. Importantly, although elevated homozygote mortality could account for the overall enrichment of *Bb* we observe among progeny, this cannot explain the much stronger disproportionate enrichment of *Bb* among large workers and gyne pupae relative to small workers (figure 6).

We did observe significantly larger size for monogyne *BB* pupae than polygyne *BB* pupae from post-treatment intervals with the highest methoprene response (figure 2A). Similarly, field observations showed that monogyne colonies (100% *BB* workers) tend to produce larger workers than polygyne colonies [71]. Nevertheless, we must emphasize that worker size varies dramatically over the course of *S. invicta* colony ontogeny [72] and that polygyne and monogyne colonies exhibit distinct patterns of queen pheromonal signalling [73,74]. Although queens were not included in any of the experimental units in our study, variation in previous pheromonal history or colony-level social context could influence how larvae integrate endocrine and nutritional cues in our experimental units. Moreover, we note that we did not detect a significant difference in growth response to JH analogue by colony social form for *BB* individuals.

Broadly speaking, two non-exclusive demographic processes could generate the observed enrichment of *Bb* among large pupae in polygyne colonies, given that the genotypes do not differ in size range: (i) loss of large *BB* larvae, or (ii) prolonged retention of large *Bb* larvae. These correspond to two developmental scenarios: differential survival during larval development, or developmental dynamics that increase the probability that *Bb* individuals attain large size. Below, we consider several non-mutually exclusive hypothetical mechanisms that could give rise to one or both of these scenarios, as avenues for future research.

Within the colony social context of *S. invicta*, genotype bias in larval survival and growth can be mediated by worker care. At the adult stage, the fire ant social supergene is known for its greenbeard effects [14,75], whereby *Bb* workers recognize and preferentially support queens carrying the same supergene haplotype but cull *BB* sister gynes with increasing frequency as they mature as adults [5,32,76]. In principle, similar greenbeard effects could induce discrimination by *Bb* workers against *BB* larvae since the size of an individual larva and strength of its hunger cue appear to be associated with the amount of feeding received by trophallaxis from adult workers in *S. invicta* [77]. In opposition to this hypothesis, enrichment of *Bb* among gyne pupae was observed in a previous experiment where all adult workers rearing the larvae had the *BB* genotype [12], making it unlikely that worker-mediated greenbeard discrimination can alone explain the enrichment of *Bb* observed in large size classes of adults produced by polygyne colonies. Nevertheless, we cannot rule out discrimination by nestmate workers as a potential factor influencing size-by-genotype interaction during larval development.

Effects of maternal genotype also have the potential to differentially influence the development of *BB* and *Bb* embryos. In polygyne *Formica selysi*, maternal effects manifest through genomic incompatibilities between eggs, which are all provisioned by queens carrying the polygyne-limited *P* supergene haplotype, and embryos that lack this *P* haplotype (*MM* homozygotes), which fail to eclose from the egg case [6,10]. Although no comparable maternal effects killing is evident in *Solenopsis*, interactions between *b-*encoded cytoplasmic factors in eggs and *BB* embryonic factors could impact developmental potential. In this manner, maternal effects could feasibly contribute to a bias in growth trajectories or caste outcomes by supergene genotype in *S. invicta*, but this has yet to be assessed empirically.

Besides extrinsic maternal and social factors, intrinsic differences between *BB* and *Bb* larvae could contribute to patterns of size and caste allocation. In holometabolous insects, body size at pupation can be considered the product of growth rate and the duration of larval development, as mediated by the critical size for moulting [78]. In caste-polyphenic systems such as ants, thresholds governing the transition to pupation (and/or early developmental commitment to reproductive caste development) could be influenced by small, temporally restricted differences in endocrine sensitivity that alter the likelihood an individual continues feeding versus committing to moult. Under this scenario, *Bb* larvae may be slightly more likely than *BB* to delay moulting owing to physiological differences, resulting in prolonged residence in key larval stages for a subset of *Bb* individuals, which could in turn increase the retention of *Bb* in larger size classes.

We tested whether such heterochrony can generate biased size representation using a stage-structured developmental simulation that allows growth trajectories between genotypes to vary (see the supplementary material for details). Two illustrative conditions are sufficient to generate the observed enrichment of *Bb* among large workers and gynes: *Bb* larvae require a longer growth window to attain the same degree of weight gain as *BB* larvae (model A), or larvae have an increased probability of extending development to a larger size caste (model B). In other words, enrichment of *Bb* among larger individuals can occur through probabilistic effects on developmental heterochrony, without requiring uniform shifts toward increased growth rate or critical size. Meanwhile, if size is an axis for caste determination [36], a modest *Bb* bias towards a caste with larger body size (model B) is sufficient to explain *Bb* enrichment among gynes without invoking a separate caste-specific mechanism. Consistent with this, the proportion of *Bb* increases with body size across both worker and gyne castes along a shared size axis (figure 4). This pattern suggests that the supergene acts on a common size-linked developmental trajectory, with enrichment in both large workers and gynes emerging as a unified downstream consequence.

The absence of a significant genotype-specific growth response to JH in our study suggests that large differences in JH sensitivity by genotype do not explain the enrichment of *Bb* in large workers and gynes. However, we observed greater overlap than expected by chance between genes with elevated expression in *Bb* adult tissues in *S. invicta* and orthologues with elevated expression in gyne-destined and JH-treated larvae in *M. pharaonis*. This highlights transcriptional concordance between effects of supergene genotype in adults and putatively conserved developmental caste trajectories that could be shaped in part by *cis*-regulatory evolution of genes residing in the supergene. Thus, our results are consistent with direct or indirect supergene effects [55] on downstream growth and caste-regulatory pathways that may partially overlap with evolutionarily conserved JH-responsive networks. In line with this hypothesis, we identified one gene with constitutively elevated expression in *Bb* relative to *BB*, *b-*allele-specific expression and regulatory element divergence in *S. invicta*, as well as elevated expression across 3rd instar larval development for gynes relative to workers and JH-responsive expression in *M. pharaonis*. This gene, *growth arrest specific 8 (gas8)*, could feasibly play a role in supergene effects on developmental trajectories, although the role of *gas8* on caste development has yet to be functionally validated. Other candidate factors for effects on size-based caste allocation in *S. invicta* include previously identified supergene-linked copy number variants that are orthologues of the body size-associated *Drosophila melanogaster* transcription factor encoding genes *cap‘n'collar* (*cnc* [79]) and *bitesize* (*btsz* [80]) [55,57]. These results highlight that supergenes may bias complex life-history traits through targeted gene regulatory changes. Further study in *S. invicta* and comparison to other supergene systems will help clarify whether endocrine modulation of developmental trajectory and timing represents a general strategy by which supergenes promote their own transmission.

## Supplementary Material



## Data Availability

All relevant data and code can be found in the supplementary material [81].
