# Supplementary material for: Developmental bias of body size and caste by the fire ant social supergene
Source: Proc Biol Sci. 2026 Oct 7;293(2080):20261338. doi: 10.1098/rspb.2026.1338 (PMC13641908; doi:10.1098/rspb.2026.1338)
Supplement: Supplementary file 1 [file rspb.2026.1338.zip › rspb20261338_si_001.zip!/Zeng et al_JH Supplementary Document_Proc B Aug19.docx]

**Supporting Information for**

**Developmental bias of body size and caste by the fire ant social supergene**

**Supplementary Text**

**Ant collection and rearing details**

Queenright and mature colonies of *Solenopsis invicta* representing both social forms were collected from Clarke and Oconee Counties, Georgia, USA, in the spring of 2024. Colonies were excavated with soil into 5-gallon buckets. Ants were separated from the soil using a drip-flotation method [1] and transferred to plastic rearing trays. The inside walls of trays were coated with PTFE (Sigma-Aldrich, Cat# 665800, dried coating of 20% dispersion in H_2_O), an anti-traction barrier to prevent escape. Each tray contained several artificial ant nests, which are plastic Petri dishes with plaster bottoms and blackened covers (d = 14 cm). The colonies were housed in an insectary maintained at 31 ± 1 °C for optimal growth [2]. Colonies were provided with continuous access to water via glass tubes fitted with cotton stoppers. The nests were watered twice a week (i.e., on Monday and Friday) and fed three times a week (i.e., on Monday, Wednesday, and Friday) with a rotating diet consisting of rehydrated freeze-dried insects as well as high-sugar and high-protein diets. Initial determination of the social form of each colony was based on the presence of more than one dealate (wingless) reproductive queen, which is diagnostic for polygyny, or a single physogastric egg-laying queen, which is typical in monogyne colonies.

**Adult gyne genotype frequency**

To estimate genotype frequency in adult gynes within polygyne colonies, we sampled young individuals to negate any potential genotypic culling bias, since most BB alate gynes are executed by polygyne workers once they reach over one week of age. We extracted gyne pupae from polygyne colonies and placed them into pre-established colony fragments for rearing. These fragment colonies were then checked every two to three days for newly eclosed adult gynes; any adult gynes found were immediately collected and genotyped. We collected 141 adult gynes from 5 polygyne colonies, 91 of them being *Bb* and 50 being *BB*.

**Ant diet receipt**

Fire ant colonies in the lab were provided with water and 3 blends of food consisting of a high-protein diet, a high-carbohydrate veggie diet, and freeze-dried insects blended with water (all foods were blended or hand mixed thoroughly into a smoothie).

The recipes of the two diets are as follows:

1. High-protein diet

- Wet dog food – 3 cans (Pedigree®, ground meet, beef or chicken based, 13.2 oz per can)
- Salmon in water – 5 cans (any brand, 5 oz per can)
- Tuna in water – 8 cans (any brand, 5 oz per can)
- Peanut butter – 1 cup (Jif®)
- Water – 1 cup

2. High-carbohydrate diet

- Gerber® Fruit baby food – 10 containers (2 oz per container)
- Gerber® Veggie baby food – 10 containers (2 oz per container)
- Mashed potato flakes – 1/2 cup
- Sugar – 2 cups
- Peanut butter – 1 cup (Jif®)
- Water – 1 cup

**Experimental procedure details**

We established experimental colonies of queenless workers and brood from either monogyne (single queen) or polygyne (multiple queens) social forms in individual plastic trays (40 × 25 cm), each containing a circular plaster nest (10 cm diameter) and a water tube for humidity maintenance (Fig. 1A). Each colony received 1.5 g of workers (approximately 2,500 individuals) and 1.5 g of mixed brood, both obtained from a distinct, mature queenright source colony (hereafter referred to as the “source colony”).

For each social form, a matched pair of acetone-control and methoprene-treated colonies was established on the same day, using brood and workers from the same source colony. Methoprene treatment (Sigma-Aldrich, catalog no. 33375, CAS No. 40596-69-8) was administered one day after colony setup via the diet. Specifically, methoprene was infused into the high-sugar diet (diet first diluted 1:1 with water to increase liquidity and facilitate uptake by workers) and given to the colony once on the day after setup. This feeding approach allowed methoprene to reach all developing larvae via workers, to preserve the natural social context for brood development, and to more closely mimic the physiological dynamics of endogenous juvenile hormone elevation. We treated mixed-stage brood because caste determination in *S. invicta* may occur early in larval or embryonic development, and effective manipulation therefore requires exposure across larval instars as well as in eggs.

Methoprene-infused diet was prepared by aliquoting 10 mL portions of sweet diet into Falcon® tubes, each supplemented with 1 mg methoprene dissolved in 10 μL acetone (final concentration in diet: 0.1 mg/mL methoprene). Control diets received 10 μL of pure acetone. All tubes were gently agitated to ensure uniform mixing. One day after colony setup, treatment colonies were given 2 mL of methoprene-infused diet, while control colonies received 2 mL of acetone-control diet to account for any potential effects of the solvent. All experimental colonies were maintained under standardized rearing conditions as previously described and were subsequently fed ad libitum with untreated alternating diets for the remainder of the experiment.

To thoroughly assess developmental trajectories under methoprene treatment, we varied the number of days between treatment initiation and pupal collection, which was defined as the “post-treatment interval,” including 15, 16, 20, 21, 22, 23, and 24 days. We included an additional polygyne pair for a 25-day post-treatment interval to receive more extra-large worker pupae for comparative analysis within the polygyne social form.

On the day of worker pupal collection, nests were transferred to temporary trays and chilled at 4°C for 30 minutes to immobilize workers and prevent them from grasping brood items with their mandibles, which would otherwise make pupal separation difficult. From each polygyne unit, we collected 96 worker pupae (except for 24-day and 25-day methoprene-treated units, where fewer pupae remained). The pupae were individually weighed and then stored at -80°C for subsequent genotyping. From each monogyne unit, 50–60 worker pupae were collected and weighed. We also weighed 30 haphazardly selected worker pupae from each corresponding Source Colony on each collection day.

Pink-eyed pupae of gynes were removed from the units as they appeared (when we inspected the nest on Monday, Wednesday, or Friday), from as early as 19 days to as late as 51 days after treatment. All gyne pupae were weighed, and those from polygyne colonies were stored at -80°C for later DNA analysis.

**Genotyping details**

Nuclear DNA was extracted from the whole bodies of selected samples using the *Quick*-DNA Tissue/Insect Microprep Kit (cat# D6015, Zymo Research), following the manufacturer’s instructions. A multiplex PCR was used to amplify the *Gp-9* locus, which is a known marker for the social supergene [3]. Some samples were processed using newly developed primers diagnostic of supergene genotype from [4]. In total, we genotyped 1049 worker pupae and 195 gyne pupae from polygyne-derived experimental units (monogyne-derived experimental units did not require genotyping, as all individuals are *BB*).

**Gene expression analysis details**

Previously, we performed integrative analyses [5] of *S. invicta* chromatin accessibility (ATAC-seq) in *BB* and *Bb* adult worker brains, gene expression (RNA-seq) in adult gyne ovaries and brains [6] and adult worker brains and gasters [7], *B* and *b* SNP allele frequencies [8], and transcription factor binding motifs [5]. Source datasets and detailed methods are described in full in Jones et al. [5]. This approach provided information on the effects of supergene evolution on gene expression, chromatin accessibility, and gene regulatory element divergence. We were further interested in comparing these data to developmental patterns of gene expression affiliated with the differentiation of body size and caste, as well as the regulatory response to methoprene treatment, but such data are currently unavailable for *S. invicta*. Fortunately, an emerging model of ant development, *Monomorium pharaonis*, belongs to the same subfamily (Myrmicinae) as *S. invicta* and has been subject to an examination of methoprene-responsive and caste-biased gene expression during larval development [9]. *M. pharaonis* developmental methoprene treatment and RNA-seq analyses are described in full detail in Li et al. [9].

We identified orthologs between *S. invicta* and *M. pharaonis* using a BLAST [10] reciprocal best hit approach with the aim of examining overlap between sets of genes with (i) evidence of caste-biased or JH analog-responsive gene expression during larval development in *M. pharaonis*, and (ii) evidence of gene regulatory evolution and differential expression in multiple tissues of adult *S. invicta.* We obtained genome annotation files from NCBI RefSeq for both *S. invicta* (GCF_016802725.1) and *M. pharaonis* (GCF_013373865.1). Using the longest isoform protein files from both annotations, we performed a reciprocal blastp (using default parameters other than a more stringent e-value threshold of10e-5) and identified 9884 pairs of genes for which searches in both directions resulted in the same hit pair (Table S2). These putative ortholog pairs were used to compare gene expression between the two species. Using *S. invicta* gene expression data first published in [6] and [7] and later integrated in [5], we first identified genes that were supergene-biased in expression (i.e., *Bb*>*BB* in four contexts: worker brains, worker gasters (abdomen), gyne brains, and gyne ovaries [all adults]) (Table S3). We next tested for overlap of these supergene-linked genes with genes identified as gyne-biased (relative to workers) or responsive to JH analog treatment (relative to controls) during larval development. From data published in [9], we extracted genes for which expression was gyne-biased in all three larval stages tested (early, middle, and late 3^rd^ instar [the final larval instar of *M. pharaonis*]), or JH-biased in at least one of the three larval stages tested (since no genes were consistently affected by JH across all 3^rd^ instar stages). We tested the significance of resulting overlaps using the SuperExactTest package in R [11,12], with a universe size of 9231 corresponding to all genes included in gene expression tests in both [5] and [9], and among the putative orthologs identified via reciprocal best hit blast. Lists of DEGs for each included pairwise comparison are in Table S3, and results of overlap tests are provided in Table S4.

**Models of genotype-dependent developmental dynamics**

To evaluate whether modest, biologically plausible differences in developmental timing are sufficient to generate the size-dependent genotype patterns observed in our data, we implemented two simple stage-structured stochastic simulation models of worker development. These models were not intended to reproduce the full physiological complexity of ant ontogeny, but rather to test the logical sufficiency of alternative developmental mechanisms proposed in the Discussion. The Python scripts used to implement the developmental simulations are included in the electronic supplementary material.

In both models, each agent represents an individual carrying either the *BB* or *Bb* genotype and progresses through three developmental stages (i.e., egg, larva, and pupa) tracked in discrete daily time steps. Stage durations are measured in days and parameterized to approximate empirically realistic developmental time scales for *Solenopsis invicta*. Egg development is fixed at five days. Larval development has a baseline mean duration of 18 days for *BB* individuals, whereas *Bb* individuals are assigned a varied expected larval duration scaled by a genotype-specific multiplier (Bb_duration) in the first model. Realized larval durations are drawn stochastically around these genotype-specific means, introducing individual-level variation in developmental timing.

Eggs are produced continuously with equal initial genotype frequencies (50% *BB* and 50% *Bb*), consistent with Mendelian expectations for offspring produced by *Bb* queens. By varying only genotype-specific larval duration and the efficiency with which developmental time is converted into mass, while holding other aspects of development constant, the models test whether heterochronic differences in developmental timing alone are sufficient to generate enrichment of Bb among larger individuals without invoking changes in critical size thresholds, caste-specific growth programs, or novel developmental mechanisms.

Body mass is accumulated during the larval stage and fixed at pupation. Rather than modeling linear daily mass gain, final pupal mass is represented as a nonlinear function of total larval duration, reflecting the empirical observation that a disproportionate fraction of biomass accumulation occurs late in larval development in holometabolous insects. Specifically, pupal mass is modeled as an exponential function of realized larval duration normalized by a genotype-specific reference timescale. For *BB* individuals, pupal mass is given by

w_BB = exp( 0.5 * ((L_BB_ / 18) - 1 ))

Where:

- w_BB = final pupal weight of a *BB* individual
- L = realized larval duration (in days)
- 18 = baseline mean larval duration for *BB*
- 0.5 = sensitivity parameter controlling how strongly duration affects weight

Departures from this baseline differ among models and are restricted to genotype-specific properties of *Bb* individuals. In all cases, *Bb* larvae are allowed to deviate from *BB* development through changes in developmental timing, growth efficiency, or the probability of extending larval development into larger size classes, while other aspects of development are held constant. The specific mechanisms by which *Bb* development diverges from the *BB* baseline differ across models and are described separately below.

*Model A: Prolonged development with reduced growth efficiency*

In the first model, *BB* and *Bb* individuals differ in developmental duration but not in caste fate, as only a single caste is present. *Bb* larvae experience a longer larval period on average than *BB* larvae, but gain mass less efficiently, such that they do not attain a higher maximal size. This is implemented by extending the larval duration of *Bb* individuals while applying a genotype-specific weight scaling factor that reduces the efficiency of weight gain.

For *Bb* individuals, pupal weight is determined using the same functional relationship, but larval duration is normalized by the genotype-specific extension of development. Specifically, *Bb* pupal weight is given by

w_Bb = exp( 0.5 * ((L_Bb_ / (18 * Bb_duration)) - 1 ))

Where:

- w_Bb = final pupal weight of a *Bb* individual
- L = realized larval duration (in days)
- Bb_duration = genotype-specific multiplier (> 1) that extends larval development in *Bb*

*Bb* pupal weight follows the same functional relationship as *BB*, while *Bb* larvae are allowed to develop for a longer period, but this extended duration does not directly translate into increased growth efficiency. As a result, they are more likely to be present in the population when sampling focuses on larger individuals, producing an apparent enrichment of *Bb* among higher weight classes (Fig. S-A). Importantly, this enrichment arises despite substantial overlap in the size distributions of *BB* and *Bb* pupae and without increasing the maximal size achieved by *Bb* individuals.

**Figure S-A.** **Simulation results under Model A: prolonged development with reduced growth efficiency.** (A) Temporal dynamics of genotype frequencies across developmental stages (larva, pupa, adult) in a stage-structured stochastic simulation. Although *BB* and *Bb* begin at equal frequencies, *Bb* individuals become progressively enriched among later developmental stages due to extended larval duration. (B) Histogram of simulated pupal weights by genotype. *BB* and *Bb* distributions largely overlap, and *Bb* individuals do not attain greater maximal size. (C) Kernel density estimates of pupal weight confirm substantial overlap in size distributions between genotypes. (D) Proportion of *Bb* individuals across pupal weight classes. *Bb* enrichment increases with body size despite similar overall weight distributions.

*Model B: Genotype-biased entry into prolonged growth trajectories*

The second model explicitly incorporates size-based worker subcastes. During the larval stage, individuals are probabilistically assigned to either a minor or a major developmental trajectory. Major development is 1.5 times longer than minor development, as defined by the *MAJOR_GROWTH_FACTOR* parameter. In this model, *BB* larvae have a 20% probability of entering the major-worker trajectory, whereas *Bb* larvae have a 35% probability.

Major workers experience a prolonged larval period and attain substantially larger size at pupation. In this model, *BB* and *Bb* individuals have identical baseline growth rates and larval durations within each trajectory; however, *Bb* individuals have a slightly higher probability of entering the major-worker (or gyne) trajectory than *BB* individuals.

This probabilistic shift alone is sufficient to generate an increase in *Bb* frequency with size, enrichment of *Bb* among large individuals, and depletion of *Bb* among minor workers, even though *BB* and *Bb* overlap extensively in body size and share the same maximal size within each caste (Fig. S-B). Thus, a modest genotype-dependent bias in developmental thresholds can produce the observed genotype–size patterns without requiring uniform changes in growth rate, critical size, or caste-specific growth programs.

**Figure S-B. Simulation results under Model B: genotype-biased entry into prolonged growth trajectories.** (A) Temporal dynamics of genotype frequencies across developmental stages (larva, pupa, adult) in a stage-structured stochastic simulation incorporating size-based worker trajectories. (B) Histogram of simulated pupal weights by genotype showing bimodal distributions corresponding to minor and major developmental pathways. (C) Kernel density estimates confirm substantial overlap in body size between *BB* and *Bb* within each trajectory. (D) Proportion of *Bb* individuals across pupal weight classes increases with size.

*Interpretation*

Together, these models demonstrate that the empirical patterns observed in this study can arise from subtle, genotype-dependent differences in developmental timing or threshold dynamics operating within a socially regulated developmental system. The models are intended to establish plausibility rather than provide parameter estimates, and they support the interpretation that the selfish bias of the *Sb* supergene may emerge from probabilistic developmental effects rather than deterministic shifts in size or caste fate.

**Supplementary Figures**

**Figure S1.** Unusually large workers from methoprene-treated colonies that resembled intercastes, in comparison to polygyne queens and the largest workers from a mature monogyne colony. Some of these intercastes develop wings (enlarged in the color image at right).

**Fig. S2.** Histograms of polygyne worker pupal weight by treatment, genotype, and size classes. Weights are pooled from experimental colonies across all post-treatment intervals of 15–25 days. For *bb*, all individuals from these polygyne colonies are shown regardless of size class.

**Fig. S3. Proportion of *Bb* worker pupae by weight quartile and treatment.** Colony-level percentages of Bb individuals among worker pupae in the bottom (first) and top (fourth) weight quartiles under acetone-control (grey) and methoprene-treated (red) conditions. Each point represents a colony; boxplots show median and interquartile range.

**Reference**

1.	Banks WA, Lofgren CS, Jouvenaz DP, Stringer CE, Bishop PM, Williams DF, Wojcik DP, Glancey BM. 1981 *Techniques for collecting, rearing, and handling imported fire ants*. Agricultural Research (Southern Region), Science and Education Administration, US Department of Agriculture.

2.	Tschinkel WR. 2006 *The Fire Ants*. Harvard University Press.

3.	Valles SM, Porter SD. 2003 Identification of polygyne and monogyne fire ant colonies (Solenopsis invicta) by multiplex PCR of Gp-9 alleles. *Insectes soc.* **50**, 199–200. (doi:10.1007/s00040-003-0662-8)

4.	Béniguel S, Hung T-T, Chen M-E, Wang J. 2025 Fire ant social supergene genotyping using polymorphic indel markers. *Insect. Soc.* (doi:10.1007/s00040-025-01047-9)

5.	Jones BM, Waugh AH, Catto MA, Kay S, Glastad KM, Goodisman MAD, Kocher SD, Hunt BG. 2025 The Fire Ant Social Chromosome Exerts a Major Influence on Genome Regulation. *Mol Biol Evol* **42**, msaf112. (doi:10.1093/molbev/msaf112)

6.	Arsenault SV, King JT, Kay S, Lacy KD, Ross KG, Hunt BG. 2020 Simple inheritance, complex regulation: Supergene-mediated fire ant queen polymorphism. *Molecular Ecology* **29**, 3622–3636. (doi:10.1111/mec.15581)

7.	Arsenault SV, Riba-Grognuz O, Shoemaker D, Hunt BG, Keller L. 2023 Direct and indirect genetic effects of a social supergene. *Molecular Ecology* **32**, 1087–1097. (doi:10.1111/mec.16830)

8.	Martinez-Ruiz C, Pracana R, Stolle E, Paris CI, Nichols RA, Wurm Y. 2020 Genomic architecture and evolutionary antagonism drive allelic expression bias in the social supergene of red fire ants. *eLife* **9**, e55862. (doi:10.7554/eLife.55862)

9.	Li R *et al.* 2024 Juvenile hormone as a key regulator for asymmetric caste differentiation in ants. *Proceedings of the National Academy of Sciences* **121**, e2406999121. (doi:10.1073/pnas.2406999121)

10.	Altschul SF, Madden TL, Schäffer AA, Zhang J, Zhang Z, Miller W, Lipman DJ. 1997 Gapped BLAST and PSI-BLAST: a new generation of protein database search programs. *Nucleic Acids Res* **25**, 3389–3402. (doi:10.1093/nar/25.17.3389)

11.	Wang M, Zhao Y, Zhang B. 2015 Efficient Test and Visualization of Multi-Set Intersections. *Sci Rep* **5**, 16923. (doi:10.1038/srep16923)

12.	R Core Team. 2021 R: A language and environment for statistical computing.
